# Cost of treatment for head and neck cancer in India

**DOI:** 10.1371/journal.pone.0191132

**Published:** 2018-01-11

**Authors:** Akashdeep Singh Chauhan, Shankar Prinja, Sushmita Ghoshal, Roshan Verma, Arun S. Oinam

**Affiliations:** 1 School of Public Health, Post Graduate Institute of Medical Education and Research, Chandigarh, India; 2 Department of Radiotherapy, Post Graduate Institute of Medical Education and Research, Chandigarh, India; 3 Department of Otolaryngology, Post Graduate Institute of Medical Education and Research, Chandigarh, India; Scientific Institute of Public Health (WIV-ISP), BELGIUM

## Abstract

There are no published data on the cost of cancer treatment for guiding reimbursement decisions in India. The present study was designed to estimate the cost of treating head and neck cancer (HNC) with the aim of determining package rates. The present study was undertaken in the Departments of Radiotherapy and Otolaryngology of a large tertiary care hospital in North India. Economic health system costs incurred were assessed using a bottom-up methodology. Data on all resources–capital or recurrent, incurred on the delivery of HNC treatment were collected from April 2014 to March 2015. Following the cost-of-illness approach, patients were interviewed to elicit out-of-pocket (OOP) expenditure. A total of INR 40,993,017 (*USD* 0.67 million) was spent on radiotherapy care for treating HNC during 1 year. Salaries constituted the major component (42.6%) of this cost, followed by equipment/furniture (29%), space rent (20.7%), overheads and consumables (7.7%). In addition, INR 47,191 (*USD* 773) per HNC patient was spent on the surgery. Furthermore, patients spent an average amount ranging from INR 12,575 (*USD* 206) to INR 65,257 (*USD* 1069) on the different treatment therapies. In terms of package rates, cobalt radiotherapy alone was the cheapest (INR 38,714, *USD 634*), while intensity modulated radiotherapy (IMRT) was most expensive (INR 192,914, *USD* 3161). The estimates from the present study could be used for developing package rates under various publicly financed health insurance schemes as well as for the planning for creation of new cancer centres.

## Introduction

India is home to 17% of the global population. On the contrary, cancer mortality due to head and neck cancers (HNC) in India constitutes nearly 28% of the world’s mortality and 71% of the mortality in the South East Asia region. [1] The rising demand for cancer care, along with the rising cost of treatment due to the introduction of several new treatment technologies [2, 3] has imposed a challenge to the health system in India.

The budget allocation towards cancer-specific spending in India has increased from INR 115 million in the sixth plan (1980–1985) to INR 28,719 million and INR 60,000 million in eleventh and twelfth five-year plan, respectively. [4–6] Furthermore, since 2007, a large amount of money has been pooled towards cancer care by the Government of India through various publicly sponsored health insurance schemes. [7–9] Also, certain states such as Punjab provide cashless cancer treatment in various public and private sector hospitals. [10] Despite this major spending on cancer, which includes large scale purchasing of care from the private sector, there are no published data on the cost of providing cancer care. Moreover, there is a significant technological advancement in the field of radiotherapy for cancer treatment. While the advanced forms of treatment, i.e., intensity modulated radiotherapy (IMRT) and 3-dimensional conformal radiotherapy (3-DCRT) [11–13] have been reported to provide better outcomes in terms of sparing of normal adjacent tissues [14, 15], there is no systematic attempt in assessing the cost of providing treatment by using these alternative technologies.

In the context of India, there are no studies which have assessed the health system cost of provision of radiotherapy or surgery related to treatment of HNC. Further, there have been only few studies estimating out-of-pocket expenditure on cancer care,[16–18] but these studies were done on data collected almost a decade back. Furthermore, most of the decisions related to reimbursement under various health insurance programs (such as Central Government Health Insurance scheme, Rashtriya Swasthiya BimaYojana etc.) in India were based on expert opinion and not on any formal costing analysis. Considering this, the present study was designed to estimate the cost of providing treatment for HNC patients by using various technologies ranging from surgery to radiotherapy (2-DRT, 3-DCRT or IMRT) or chemotherapy, as well as for different combinations of these. This would finally lead to designing of provider payment package rates for the various treatment options.

## Material and methods

The present study was part of a broader study evaluating cost-effectiveness of two radiotherapy modalities, i.e., 2-dimensional radiotherapy (2-DRT) versus intensity modulated radiotherapy (IMRT) for treating HNC in the context of India. The protocol of the broader study is deposited as laboratory protocol in protocols.io with DOI link: dx.doi.org/10.17504/protocols.io.jzvcp66.

### Study setting

The present study was undertaken in the Radiotherapy and Otolaryngology departments of the Postgraduate Institute of Medical Education and Research (PGIMER), a tertiary care institute located in Chandigarh, India. With regards to cancer treatment, there is a provision of surgical care, chemotherapy and radiotherapy. Specifically, the radiotherapy department has 10 oncologists, 23 resident doctors, 6 medical physicists and 27 technical staff members (senior and junior technicians) and 5250 patients received radiotherapy either alone or with chemotherapy during 2014–15. Six radiotherapy machines, 2 using Cobalt-60, 4 using linear accelerators (2 Low energy 6 MV x-ray machines (DBX), 1 Dual high energy (6 MV and 15 MV) x-ray machine (DHX) and 1 image guided radiotherapy machine (IGRT) of high energy x-ray (6 MV and 15 MV)), 2 CT simulators and one conventional simulator were used for providing treatment.

### Flow of treatment process

Patients suspected of having HNC are first consulted in the outpatient clinic of the Otolaryngology Department. The patient undergoes all the diagnostic tests for the final diagnosis. If required, surgery is done by the surgeon of the Otolaryngology Department. If a patient requires radiotherapy or chemotherapy (with or without surgery), he/she is referred to the Radiotherapy Department

### Data collection

#### Health system costing

As the main objective of the study was to calculate the unit cost of the various treatment options for cancer care, cost data specifically for surgical care were calculated for a single unit consisting of an ENT surgeon and his team. On the contrary, data were collected for the entire Department of Radiotherapy.

Health system costs were assessed following the economic costing and bottom-up methodology.[19, 20] Data collection tool was adapted from the previous studies done in similar settings (S1 File). [21, 22] The first step for the cost assessment was to classify cost centres. Outpatient department, inpatient ward, operation theatre and radiotherapy complex were classified as direct cost centres. Laboratory, pharmacy and radio diagnosis complex were categorised as indirect cost centres. Specifically for radiotherapy, cost centres were further divided based on the activities, i.e., CT simulation, dosimetry/planning, quality assurance, preparation and delivery of radiotherapy.

Data on both the capital and the recurrent resources spent on the delivery of cancer care for the financial year April 2014 to March 2015 were collected. A facility survey was undertaken to assess the capital and physical infrastructure, i.e., building, space, furniture and equipment (Table 1). The space of each room in square feet was obtained from the Department of Hospital Engineering. Recurrent resources in the form of materials/supplies were estimated by reviewing the consumables stock register, vouchers and pharmacy records. Data on salaries, another recurrent resource, was retrieved from the salary records of all the staff members (partly or completely involved in cancer care). Service output in terms of the number of outpatient consultations, hospitalizations, surgeries, radiotherapy sessions were assessed from the routine medical records. Details on the number of various diagnostic tests prescribed to the patients were assessed from the patient files.

**Table 1:** Sources of data and apportioning statistics for estimating health system cost of HNC treatment.

Resource	Source of information	Units	Apportioning statistics
**Salaries details (basic pay, grade pay, allowance, tuition fee, dearness allowance, transport allowance, non-practice allowance, telephone allowance, learning resource allowance, etc.)**	Salary slips of each staff member from the accounts department	Indian Rupees (INR)	Proportional time spent on various activities (Time allocation interviews and observation)
**Space/Building**	Engineering department/facility survey	Square feet	Proportion of head and neck cancer patients Proportional time spent by the head and neck cancer patients.
**Furniture and Equipment (including medical and non-medical equipment)**	Facility survey/Stock registers	Number of items in working condition	Proportion of head and neck cancer patients Proportional time spent by the head and neck cancer patients.
**Drugs and consumables (masks, stereotactic frame, X-ray films, injections, stationary, sanitary & surgical supplies, office/administrative supplies, etc.)**	Consumables stock registers/Indent books/vouchers/pharmacy and store records	Number of items consumed	Based on the number of patients
**Water bills**	Water department	Expenditure in INR	Floor area
**Electricity**	Based on actual measurement of load in each room by actual assessment of electrical points	Kilowatt hour	Proportional time spent by the head and neck cancer patients.
**Laundry**	Laundry	Number of different items	Proportion of head and neck cancer patients
**Dietetics**	Department of dietetics	Number of diets served	Proportion of patients hospitalized for head and neck cancers

All the medical staff and the technical staff involved in the planning and treatment of the radiotherapy were interviewed with a semi structured interview schedule for assessing their time spent on the different services which were done either on a routine or on fixed basis (weekly, monthly, annually, etc.). Alongside these interviews, observation-based data were also collected for the time spent on regular activities. Furthermore, time spent on planning and delivery of radiotherapy treatment on the various machines (simulators, cobalt or linear accelerators) for different radiotherapy plans for a HNC patient was also observed.

The expenditures incurred towards water, maintenance, dietetics and laundry were determined from the respective departments. For assessing the space cost, estimates of the rental price (current market price) of a similar space was used. Procurement prices of the medical equipment, drugs, surgical, stationary and sanitary supplies were obtained for the year 2014–15 from the procurement department and the central store of the study hospital. For non-medical items, like furniture, market prices were used because of lack of local procurement price data. However, market prices were adjusted to reflect the hospital procurement price based on the average rates of price between the hospital procurement and the market rate for other items. The average life of the equipment and other capital resources was estimated by interviewing the personnel from the respective departments who used the items. For assessing per bed day cost in surgical department and the unit cost of various diagnostic tests, estimates from a previous study, conducted in the same hospital, were used. [22]

#### Out-of-pocket expenditure

The cost-of-illness approach classifies out-of-pocket (OOP) expenditures into direct (direct health care expenditure and direct non-health care expenditure) and indirect expenditures. [23] In the present study, only the direct OOP expenditure was assessed, as the objective was the calculation of the package reimbursement rates. Spending on diagnostic tests, radiotherapy, drugs (including chemotherapy and biologic agents), hospitalization, user fee, etc. were included under the direct health care expenditure. On the other hand, the transportation, boarding/ loading and food expenses were considered under the direct non health expenditure. A pretested semi-structured schedule, adapted from previous studies done in the similar settings, was used to interview the patients (S2 File). [21, 24]

A sample size of 410 patients was calculated based on an average weekly OOP expenditure of INR 1062, standard deviation of 412 [16] and INR 40 as the level of precision at 95% confidence interval. Data on OOP expenditure was calculated from two groups of patients. The first group comprised the patients (n = 159) who were prospectively followed up and interviewed daily till the entire duration of their radiotherapy treatment. The second group comprised the patients (n = 315) who were interviewed at the time of their first follow up or within one month of their post radiotherapy treatment. If the patient had undergone surgery, the expenditure incurred on the same part was collected retrospectively in both the groups.

### Data analysis

Capital expenditure was annualized to arrive at the equivalent annual cost taking into consideration the discount rate (time preference for money and inflation) and the lifespan of the capital equipment. [25] A five percent discount rate was used. [20, 25] For some of the staff members (oncologist, nurses), who were jointly involved in a number of activities (outpatient or inpatient care, planning or delivery), cost allocation to the particular activity were apportioned based on the proportional time contribution towards each of these activities, and then multiplying it with the gross salary of the staff member. Likewise, certain resources (capital or recurrent) which were jointly shared in two or more services, were apportioned using appropriate statistics as shown in Table 1. All the results in the present study are calculated in Indian National Rupees for the year 2014–15 and were also converted into USD (United States Dollars) based on the conversion rate of 1USD = 61.02 INR, as reported by the World Bank.

For assessing the uncertainty and variability of the cost estimates, we undertook a multivariate probabilistic sensitivity analysis in which the prices of the resources (spent on the cancer treatment) were varied using gamma and normal distributions. The base value of the salaries was varied by 20% on the upper limit to 75% on the lower limit. Base prices of radiotherapy equipment were varied by 40% on both the sides. As the prices of the drugs and the consumables show wide variation, we varied these by 100% on both the sides of the base value.[26, 27] Further, price of furniture, stationary and sanitary items were varied by 25% on both sides. Furthermore, building cost/rental prices and prices of the laboratory tests were varied by 50% on either side of the case value. Once assigning the distribution and variability to the prices of the resources, 1000 Monte Carlo simulations were run, under which different values were picked from the each of the distribution, finally coming up 1000 unit cost estimates. [28] From these 1000 unit cost estimates, an average unit cost along with 95% CI was calculated.

### Ethical consideration

The study was approved from the study hospital committee i.e., ‘Institutional Ethics Committee, Post-Graduate Institute of Medical Education and Research, Chandigarh’ with a reference number of NK/2490/Ph.D/6374. Written informed consent was obtained to interview the patients.

### Package rate estimation

Package rates of the various treatment therapies available for HNC were calculated by adding both the health system cost and OOP expenditure (direct health care expenditure). For a particular treatment alternative, the package cost consisted of all the expenditure incurred towards outpatient patient consultation, diagnostics, inpatient care (if any), and radical treatment i.e., surgery or radiotherapy or along with chemotherapy.

## Results

### Health system costing

#### Services detail

A total of 1227 patients received outpatient consultation and 225 were hospitalized for HNC in the Radiotherapy Department. In terms of the treatment undertaken, 55.4% of the cases underwent only primary radiotherapy, 29.3% had radiotherapy along with chemotherapy, 10% underwent surgery followed by radiotherapy and the remaining 5.3% had post-operative radiotherapy combined with chemotherapy. Finally, a total of 939 patients received radiotherapy, of which, 805 patients were given conventional radiotherapy (583 on cobalt machine and the rest 222 on linear accelerators), 77 patients underwent 3D-CRT and 57 patients received IMRT. In terms of site specific breakdown, 29% were oropharynx cancer, followed by cancers of glottis/supra-glottic region (19%), cancer of tongue (15%), hypo-pharyngeal cancer (14%), cancers of the nasal region including nasopharynx, nasal cavity, paranasal sinus, etc. (8%), cancer of larynx and tonsil (5%) and others (10%) including cancers of oral cavity, maxilla, soft palate, buccal mucosa, parotid glands and the lip.

#### Annual cost

A total of INR 40,993,017 (*USD* 0.67 million) was spent annually for providing HNC care in the Radiotherapy Department. Of this, 29% and 20% was spent on the delivery of 2-DRT on cobalt and linear accelerator respectively. Similarly, provision of IMRT and 3D-CRT consumed around 20% and 11% of the total cost respectively. The remaining 20% of the overall inlay was spent on the outpatient and inpatient care.

#### Input-wise distribution of annual cost

**Fig 1:**
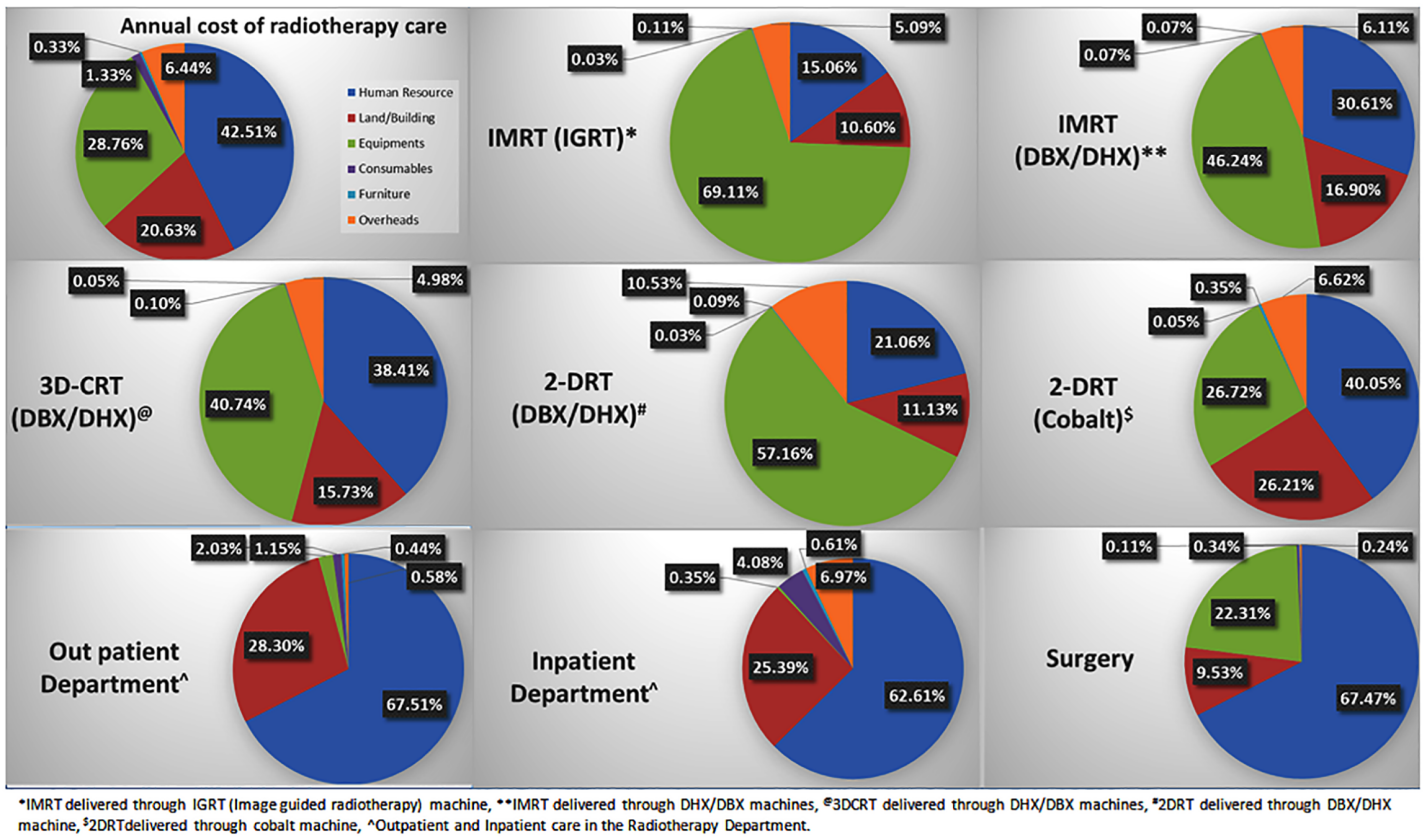
Input-wise distribution of annual health system cost of various services delivered for head and neck cancer care.

Of the overall cost on radiotherapy care, salaries constituted the major component (42.6%), followed by equipment/furniture (29%), space rent (20.7%), overheads and consumables (7.7%) as shown in Fig 1. Likewise, Fig 1 also shows input-wise distribution of the annual cost incurred on specific services i.e., outpatient and inpatient care, radiotherapy techniques and surgery. While the cost of equipment was the major component for delivering radiotherapy using IMRT (46% to 69%) and 3-DCRT (41%), salary of human resources was the single largest component in the case of cobalt radiotherapy (40%).

#### Unit costs

Per visit outpatient consultation cost was INR 302 (*USD* 5) and INR 538 (*USD* 9) in the Otolaryngology and Radiotherapy Department, respectively (Table 2). The cost of performing surgery was INR 47,191 (*USD* 773) per HNC patient. Specifically, among radiotherapy techniques, providing 2-DRT through the cobalt machine was the cheapest, i.e., INR 17,896 (*USD* 293) per patient. On the contrary, IMRT through IGRT and DBX/DHX machine was the most expensive, i.e., INR 163,728 (*USD* 2683) and INR 69,920 (*USD* 1146) respectively.

**Table 2:** Unit (health system) cost of services for the treatment of head and neck cancer.

S.No	Type of service	Unit	Unit cost in INR (95%CI)	Unit cost in USD (95%CI)
**1**	**Out-patient consultation**	Per visit cost		
	**Radiotherapy**		538 (531–546)	9 (8.70–8.94)
	**Otolaryngology**		302 (301–304)	5 (4.93–4.97)
**2**	**In- patient care (Radiotherapy department)**	Per bed day cost	3,096 (3,056–3,136)	51 (50.08–51.39)
**3**	**Diagnostics**	Per patient cost	4,108 (4,077–4,139)	67 (66.81–67.82)
**4**	**Surgery**	Per patient cost	47,191 (46,660–47,721)	773 (764.67–782.06)
**5**	**IMRT given on IGRT machine**	Per patient cost	163,728 (163,22–164,229)	2,683 (2,674.95–26,91.40)
**6**	**IMRT given on DBX/DHX machine**	Per patient cost	69,920 (69,312–70.528)	1,146 (1,135.89–1,155.82)
**7**	**3D-CRT given on DBX/DHX machine**	Per patient cost	52,133 (51,640–52,626)	854 (846.28–862.44)
**8**	**2-DRT given on DBX/DHX machine**	Per patient cost	35,246 (34,945–35,547)	578 (572.69–582.55)
**9**	**2-DRT given on cobalt machine**	Per patient cost	17,896 (17,728–18,063)	293 (290.52–296.03)

### Out-of-pocket expenditure

For radiotherapy, patients spent an OOP expenditure ranging from INR 12,575 (*USD* 206) on 2-DRT to INR 21,026 (*USD* 345) on 3D-CRT to INR 21,007 (*USD* 344) on IMRT (Table 3). Similarly, OOP expenditure on surgery was INR 30,768 (*USD* 504). Further, when given in combination, direct health expenditure varied from INR 17,061 (*USD* 280) on chemotherapy combined with 2-DRT to INR 65,257 (*USD* 1069) on surgery along with IMRT and chemotherapy. Pre-hospital OOP expenditure among cancer patients seeking care at other primary or secondary care providers was INR 20,435 (*USD* 335).

**Table 3:** Per patient out-of-pocket (OOP) expenditure incurred at various levels of treatment for head and cancer.

Treatment Options	Per patient OOP expenditure (Including direct non-health expenditure)		Per patient OOP expenditure, (Excluding direct non-health expenditure)	
	INR (95%CI*)	USD (95%CI*)	INR (95%CI*)	USD (95% CI*)
**Surgery**	44,098 (33,859–53,837)	723 (555–882)	30,768 (22,070–39,712)	504 (362–651)
**2-DRT**	31,487 (29,123–33,893)	516 (477–555)	12,575 (11,485–13,713)	206 (188–225)
**Chemotherapy + 2-DRT**	35,471 (31,304–39,945)	581 (513–655)	17,061 (14,874–19,462)	280 (244–319)
**Surgery + 2-DRT**	52,595 (38,179–68,615)	862 (626–1124)	32,650 (21,475–44,953)	535 (352–737)
**Surgery + 2-DRT + chemotherapy**	57,873 (42,096–78,090)	948 (690–1280)	32,595 (18,722–49,762)	534 (307–816)
**3DCRT**	40,377 (22,234–60,333)	662 (364–989)	21,026 (10,955–31,466)	345 (180–516)
**Chemotherapy + 3dcrt**	47,468 (31,470–64,134)	778 (516–1051)	25,675 (16,089–37,156)	421 (264–609)
**Surgery + 3dcrt**	58,824 (24,553–1,03,111)	964 (402–1690)	37,213 (15,513–69,630)	610 (254–1141)
**Surgery + 3DCRT + Chemotherapy**	78,376 (24,950–1,14,770)	1,284 (409–1,881)	56,351 (14,450–97,770)	923 (237–1,602)
**IMRT**	42,405 (33,008–52,604)	695 (541–862)	21,007 (15,623–26,891)	344 (256–441)
**Chemotherapy + IMRT**	47,995 (37,237–58,621)	787 (610–961)	26,029 (19,629–32,929)	427 (322–540)
**Surgery + IMRT**	76,262 (59,156–93,961)	1,250 (969–1,540)	53,619 (41,386–66,633)	879 (678–1,092)
**Surgery + IMRT + chemotherapy**	86,692 (56,229–1,18,851)	1,421 (921–1,948)	65,257 (35,658–97,380)	1,069 (584–1,596)

*CI-Confidence interval

### Package rates

Among various treatments when given alone, provision of 2-DRT from the cobalt machine came out to be cheaper (INR 38,714; *USD 634*) and IMRT given through IGRT machine was the costliest, i.e., INR 192,914 (*USD* 3161). When multiple treatment therapies are provided, package rates of these various combinations are shown in Table 4.

**Table 4:** Package rates (INR) of various treatment options for treating head and neck cancer in India.

Types of radiotherapy	Whether surgery or chemotherapy given			
	No surgery or chemotherapy	Surgery	Chemotherapy	Both surgery and chemotherapy
**No Radiotherapy**		102,313 (101,697–102,929)		
**2DRT (Cobalt)**^@^	38,714 (38,500–38,926)	124,880 (124,059–125,700)	43,274 (43,048–43,450)	125,004 (124,134–125,873)
**2DRT (DBX)**^#^	56,064 (55,738–56,390)	142,230 (141,339–143,122)	60,624 (60,290–60,958)	142,354 (141,418–143,290)
**3DCRT (DHX/DBX)**^$^	81,594 (80,983–82,205)	163,729 (162,324–165,135)	86,193 (85,470–86,917)	166,594 (164,775–168,413)
IMRT (DHX/DBX)**	99,107 (98,443–99,771)	198,075 (196,947–199,204)	104,288 (103,618–104,958)	209,777 (208,291–211,263)
IMRT (IGRT)*	192,914(192,347–193,482)	291,883 (290,841–292,925)	198,095 (197,511–198,679)	303,583 (302,168–305,001)

*IMRT delivered through IGRT (Image guided radiotherapy) machine,

**IMRT delivered through DHX/DBX machines,

^$^3DCRT delivered through DHX/DBX machines,

^#^2DRT delivered through DBX/DHX machine,

^@^2DRTdelivered through cobalt machine.

Estimates in parenthesis report 95% Confidence intervals.

## Discussion

The increased demand for cancer care along with its high cost imposes a significant fiscal pressure on the government as well as financial hardships for the households.[29] Moreover, introduction of several new treatment technologies, drugs and diagnostics, have added to the cost.[2, 3] In India more than 70% of the overall health expenditure is paid out-of-pocket, which puts a huge financial burden on the citizens.[30, 31] Considering this, the Government of India pooled a large amount of money towards cancer care through various initiatives. [7–9, 32]. However, there are no empirically derived estimates of the cost for providing cancer care in India.

The present study was undertaken in a tertiary care hospital of North India to estimate the cost of treating HNC. The health system cost incurred for surgery was 47,191 (*USD* 773) per HNC patient. The cost of radiotherapy per patient varied from INR 17,896 (*USD* 293) using a cobalt machine to INR 163,728 (*USD* 2683) using IGRT machine. In terms of package rates, inclusive of OOP expenditure, treatment using cobalt radiotherapy alone was the cheapest (INR 38,714; *USD 634*) option, while intensity modulated radiotherapy through IGRT machine was the most expensive (INR 192,914; *USD* 3161).

### Case base package rate

As per our knowledge, this is the first study from India that comprehensively estimated the package rates of various therapeutic options for HNC considering both the health system costs as well as OOP expenditure. In India, treatment in public hospitals is subsidized by the government. However, some proportion of the total cost is borne OOP by the patient. [16, 24] Hence, while calculating package rates, considering both health system and OOP expenditure becomes necessary. There is a significant monopoly in the health market for tertiary care. [33] Hence, the price fixed by the private hospitals is not the true efficient price based on the economic principles of demand and supply, as it includes a significant portion of profit. Similarly, calculating only public health system cost also may not reflect the true price, as patients end up paying a significant amount as OOP expenditure on the purchase of drugs (including chemotherapy and biologic agents), consumables and diagnostics in the public hospitals.[16, 24] Therefore, in Indian settings, considering both the health system and patient perspective in public sector setting becomes obligatory while calculating package rates. The literature shows that there are no studies assessing health system cost of radiotherapy or surgical care related to HNCs. While there are few studies reporting patient level OOP expenditures on cancer care, [16–18] these do not provide a comprehensive estimate of the cost of cancer care due to the lack of estimates on health system cost.

Most of the cancer treatment in India is provided at tertiary care hospitals, as its treatment requires intensive treatment therapies which are only available at the level of these facilities. Considering this, the present study was undertaken in one of the largest public sector tertiary care hospital catering to more than 6 states, and providing outpatient and inpatient care to 2 million and 0.8 million patients each year, respectively. With regards to cancer care, more than 100 staff members are involved in its delivery and more than 5000 patients are treated annually. The presence of highly qualified medical staff, a wide spectrum of technology with high quality machinery ranging from CT scanning to intensity guided radiotherapy (IGRT), and a large patient load, justifies the appropriateness of the package rates, calculated based on the study hospital.

### Methodological issues

We used standardized costing and analytical methods for estimating the health system cost and took data for one complete year to exclude any seasonal variation of service utilization. Specifically regarding overheads, only aggregate data on resources were available. Standard apportioning statistics were used to assess the overhead cost on HNC care. However, since the overall contribution of the overhead cost on the total cost has been reported to be less than 5% in most of the Indian costing studies, [34, 35] it is unlikely to bias the overall findings.

Although the present study was undertaken in a single tertiary care institute of India, we have undertaken an extensive uncertainty analysis, based on the differential in prices of various resources i.e., salaries, cost of building, drugs, consumables and equipment across India. Based on this analysis, an average unit cost along with 95% CI was calculated for various services given for the treatment of HNC. As the quantity of resource use would remain more or less the same in tertiary care hospitals across different states in India, most of the difference in the cost estimates is due to the differential salaries and procurement prices of various resources. So, the average unit cost based on the Monte Carlo simulations is a robust estimate and the 95% CI represents the range in which cost estimates could vary across India. Nonetheless, we acknowledge undertaking single institute study as one of the limitation of the study and recommend undertaking future research studies across the country from various settings to account for variability in the level of resource use and prices. We also acknowledge that only a single unit of the Otolaryngology Department consisting of an ENT surgeon and his team was considered for the assessment of the cost of surgical care. Hence, while the unit cost of surgery and the distribution of cost is valid, we did not assess the overall annual cost on surgical care. However, these unit costs estimated are not going to bias the study results as the nature of surgeries performed between different surgeons of the department is the same. However, it is possible that practices may vary between surgeons in terms of time spent prior to surgery, during the procedure and for the post-operative care. We acknowledge this as a limitation of the study.

Expenditure paid by the patients varied from 15%-37% of the total package cost among various treatment alternatives. Most of this expenditure was spent as user fees and on the purchase of medicines, consumables and diagnostics from the market. When compared with the estimates from a study done a decade back, [16] the expenditure assessed in the present study was 3 to 7 times higher. The difference could be due to the time difference of a decade between both studies and the possibility that the inflation across these years might have increased currency values between the two time frames.

### Technology and rising costs

Our study supports the fact that introduction of newer technologies such as 3D-CRT and IMRT led to an increase in the cost of cancer care. The health system cost of treating a patient with 3D-CRT and IMRT (on IGRT) was found to be around 3 to 9 times higher than for the conventional treatment on a cobalt machine. The predominant reason for this increase in cost is the high cost of technology. While delivering conventional radiotherapy through the cobalt machine, equipment cost constitutes around 27% of the total cost, which increases to more than the double, i.e., 57%, when giving the same conventional radiotherapy through linear accelerators. This proportional cost on equipment increases to 69% for IMRT delivered through IGRT. The second and often unrecognised reason for the increase in cost is the requirement of more specialized personnel as well as more person-hours for operating these technologies. This is also reflected in the cost of personnel time spent on planning of a radiotherapy treatment. The planning cost for 3D-CRT and IMRT was 4.3 to 5.5 times higher than for 2-DRT on a cobalt machine respectively.

### Comparison with existing package rates

The existing package rates under the various health insurance programs ranging from the social health insurance schemes, i.e., the Central Government Health Insurance scheme (CGHS) [36] and Employees’ State Insurance Scheme (ESIS) [37] to the fully publicly-sponsored insurance schemes [7–9] are based on expert opinions and not on any formal costing or cost-effectiveness analysis. Table 5 compares the existing package rates for provider payments in different schemes with the actual cost reported in the present study. The rates calculated in the present study are on the lower side when compared to that under CGHS (Delhi rates), ESIS (Delhi rates) and RSBY-plus (Himachal Pradesh) except IMRT delivered through IGRT. On the contrary, these rates were on the higher side when compared with the rates of the RGJAY and RAS schemes. The lack of similarity between the existing package rates and the rates calculated in our study justifies the need for careful consideration of existing package rates.

**Table 5:** Package rates for radiotherapy for treatment of head & neck cancer across various health insurance schemes in India.

Radiotherapy techniques	Package rates under various Insurance schemes in INR (USD)					
	CGHS*	ESIS**	RSBY-plus^$^	RAS^#^	RGJAY^@^	Present study
**Cobalt 60 radiotherapy**	78,689 (1290)	70,000 (1147.5)	24,000 (393)	20,000 (328)	20,000 (328)	38,714 (634)
**2-DRT (Linear accelerators)**			60,000 (984)	50,000 (820)	50,000 (820)	56,064 (919)
**3 dimensional conformal radiotherapy**		95,000 (1557)	90,000 (1475)	75,000 (1230)	75,000 (1230)	81,594 (1337)
**Intensity Modulated radiotherapy (IMRT)**	116,010 (1902)	129,000 (2115)	120,000 (1967)	100,000 (1639)	100,000 (1639)	99,107 (1624)
**IMRT through Image guided radiotherapy machine**	169,068 (2772)	188,000 (3082)			150,000 (2459)	192,914 (3162)

*Central Government Health Insurance scheme,

**Employees’ State Insurance Scheme,

^$^RashtriyaSwasthiyaBimaYojana-plus in the state of Himachal Pradesh,

^#^Rajiv Aarogyasri Health Insurance Scheme in Andhra Pradesh,

^@^Rajiv GandhiJeevandayeeArogyaYojana in Maharashtra.

Although new technology comes with higher health benefits, it also leads to an increased cost. Studies conducted globally have shown that there is reduced incidence of side effects/toxicities with the use of 3D-CRT or IMRT as compared to 2-DRT.[14, 15] It becomes imperative on the part of the government to introduce the new treatment technology which results in more health benefit, irrespective of the cost. But because of budget constraints, it is impossible to introduce every new technology. Thus, it is necessary to undertake economic evaluations, such as, cost effectiveness analysis, which could give evidence in terms of which technology offers the best value for money spent.

## Supporting information

S1 Table Cheers checklist. (PDF)

S1 File Health system cost data collection tool. (PDF)

S2 File Out of pocket expenditure data collection tool. (PDF)
