# Supplementary material for: Cost of treatment for head and neck cancer in India
Source: PLoS One. 2018 Jan 11;13(1):e0191132. doi: 10.1371/journal.pone.0191132 (PMC5764364; doi:10.1371/journal.pone.0191132)
Supplement: S1 File — (PDF) [file pone.0191132.s002.pdf]

HEALTH SYSTEM COSTING TOOL

This tool intends to collect information pertaining to following heads for the ***financial year______:***

	**Different heads for cost data collection**	**Put tick mark at the end of interview**
**1.**	Personnel	
**2.**	Record on services delivered	
**3.**	Sources of revenue	
**4.**	Details regarding population covered and location of the facility	
**5.**	Equipment	
**6.**	Consumable drugs	
**7.**	Consumable Materials and Supplies/ Details on any other Kit	
**8.**	Physical infrastructure	
**9.**	Stationary	
**10.**	Utility/Overheads	
**11.**	Grants utilised	
**12.**	Time allocation sheet	

Table 1: Salary details

Radiation oncologist= 1, Junior resident = 2, senior resident = 3, Medical Physicist= 4, Senior technician = 5, Technical assistant = 6, Junior technician= =7, staff Nurse= 8, Radiographer= 9, Logistic Assistant= 10, Registration Clerk= 11, Statistical Assistant/ Data Entry Operator= 12, sweeper = 13.

For more than one person of a category use alphabetic prefixes. For e.g. if there are 2 Medical officers, use code: 1a, 1b

Staff No. Code	***Monthly gross salary(inclusive of all allowances or deductions)***	***How many days h/she did not work in year of data collection i.e. 2011-12(**leaves in a year)***	***Annual Incentive received for trainings (TA/DA received for trainings)***

Table 2: Details of annual allowances received (Interviews and record review)

Staff No. Code	***Government residence***		***Transport facility***		***Uniform provided/ allowance***		
	Square meter or square feet of the house building or rooms provided (Do mention the unit of data collection)	*Amount paid in a year or How much would you pay if you would rent this house i.e. monthly rental price*12?*	*Amount paid in a year*	*Vehicle name and year of make, if provided free*	*Times per year (a)*	*Unit cost of uniform (b)*	*Amount incurred on uniform (a*b) or<br><br>If unit cost not available ask, "For how much it will be available from market, if bought on its own?"*

Table 3: Annual services delivered (Record based)

**Codes**	**Services delivered**		**Services delivered during last year**
1.	*Outpatient consultations in radiotherapy department*	*Total in last 1 year*	
2.	*Outpatient consultations in ENT department*	*Total in last 1 year*	
3.	*Patients admitted for inpatient care*	*Total in last 1 year*	
4.	*Patients treated on 2DRT*	*Total in last 1 year*	
5.	*Patients treated on 3D-CRT*	*Total in last 1 year*	
6.	*Patients treated on IMRT*	*Total in last 1 year*	
7.	*Number of surgeries*	*Total in last 1 year*	
8.	*CT scans*	*Total in last 1 year*	
9.			
10.			
11.			
12.			

Table 4: Sources of Revenue

		**Amount collected during the period of data collection**
1.	*Procedure fee*	
2.	*Fee for Lab and diagnostic tests*	
3.	*Any other (specify)*	
	Total user fee from 2014-2015	

Table 5: Equipment (Observation cum record review of stock registers)

Equipment	*Quantity*	*Price*	*Expected life of equipment*	*List services for which it is used. Write serial number codes from Tables on time sheet allocation*

Table 6: Consumables. Drug (Review the stock- register and list the quantity and price of drugs in drug list sheets provided at the end of tool by using the sheets to photocopy index of drugs register and write quantity, price and utility against each drugas given below

Name of drug	*Quantity*	*Price*	*List services for which it is used. Write serial number codes from Tables on time sheet allocation*

Table 7: Consumables. Material and Supplies

Material and supplies	*Quantity*	*Price*	*List services for which it is used. Write serial number codes from Tables on time sheet allocation*
Fixation masks			
X ray films			
Simulation and portal films			

Table 8: Physical infrastructure (Interview based)

Table 12 a: Particulars	***Specify***
Area of the building (Total area in Sq. ft.) (Covered + open space)	
What is the rental price of 100 sqft place where this Public Health centre is located?	
Was there any expense on construction of building or renovation during the period of data collection	

Facility Check

Ask the head of the facility if you can make a tour of the facility to get some information on the building space, vehicles and equipment.Use the space below to draw a simple layout of the facility. Identify the type of service delivered in each room/space using the codes available. Specify a number for each room /space on the map.

Alternatively you can ask for map of the building with area measurements.

Thank the head of the facility and ask him if you can revisit the different rooms to complete measurements (*if required)* ***and make a closer observation.***
Use the following table to fill in the required information for each room in the building(s)
You need to have a measuring instrument (used to calculate length and width of the room) with you to measure square meter surface area

You need to complete the following observations:
Record the measurements needed in Table 12b
Draw a sketch of the facility in the space available above
Complete Table 13*with the inventory of furniture and items available in each room.*

Facility space

N.B.

_ ***Do not forget waiting areas*** *(some of them can be for adult only or children only).*

_ ***If any of the rooms are not used at the moment*** *indicate this in the service/ function column.*

Sketch of the facility:

Table 9: Services delivered in different rooms in facility (Put 1 if particular service is delivered in a particular room)

		Codes for Services delivered																													
Name of room	***Square meter Or feet***	***1***	***2***	***3***	***4***	***5***	***6***	***7***	***8***	***9***	***10***	***11***	*12*	*13*	*14*	*15*	*16*	*17*	*18*	*19*	*20*	*21*	*22*	*23*	*24*	*25*	*26*	*27*	*28*	*29*	*30*

Table 10: Items in facility rooms (Observation and record review)

Do ask for any items that are there in stock register and are stored due to non-utilisation or non- functionality

Name of the equipment or furniture	***Quantity of functioning items in each room***																
	Room no. 1	***Room no. 2***	***Room no. 3***	***Room no. 4***	***Room no. 5***	***Room no. 6***	***Room no. 7***	***Room no. 8***	***Room no. 9***	***Room no. 10***	***Room no. 11***	***Room no. 12***	***Room no. 13***	***Room no. 14***	***Room no. 15***	***Room no. 16***	***Corridors***
Almirah (Big steel)																	
Almirah (Small steel)																	
Almirahs (Small wooden)																	
Armless chairs																	
Bed side attendant chair																	
Bed side locker																	
Bed side Screen																	
Bed side table																	
Buckets																	

Table 11: Stationary and other miscellaneous items: (Record review for billed amounts of purchased stationary)

	**Quantity**	**Price**	***List services for which it is used. Write serial number codes from Tables on time sheet allocation***
Article indent book			
Attendance register			
Bath soap			
Carbon paper			
Cash receipt book			
Disinfectant fluids (Phenly-Ltr)			
Harpic			
Health management info system subcenter register			
Indoor register			
Nirma			
OPD card			
OPD register			
Out-station dak book			
Pencil			
Broom			
Photostat paper			
Pocha			
Poly bags for biowaste			
Register IDSP			
Savlon solution			
Spirit			
Stamp ink			
Stamp pad			
Stock + OPD register			
Toilet brush			
Towels + dusters			

A-4 paper			
Vim powder			
Article indent book			
Attendance register			
Bath soap			
Carbon paper			
Cash receipt book			
Disinfectant fluids (Phenly-Ltr)			
Harpic			
Health management info system register			
Indoor register			
Nirma			
OPD card			
OPD register			
Out-station dak book			
Pencil			
Broom			
Photostat paper			
Pocha			
Poly bags for biowaste			
Register IDSP			
Savlon solution			
Spirit			
Stamp ink			
Stamp pad			

Table 12: Utilities/Overhead (Annual)

	Quantity	***Price***	***List services for which it is used. Write serial number codes from Tables on time sheet allocation***
1.Means of transport			
Maintenance			
Repairs			
Insurance			
Others			
Total *(If available)*			
2. Building			
Electricity			
Water			
Facility rent (if relevant)			
Maintenance			
Telephone			
Kerosene			
Other			
Total *(If available)*			
3. Equipment			
Maintenance			
Repairs			
Other			
Total *(If available)*			
4. Laundry			

Table 13: Laboratory/ Radiological investigation/Procedure fees

**Type of tests**	**Quantity**	***List services for which it is used. Write serial number codes from Tables on time sheet allocation***
Haemoglobin		
TLC		
DLC		
ESR		
Blood grouping		
Bleeding time, clotting time (BT and CT		
Blood sugar		
**Diagnostic test (General)**		
X-rays		
Ultrasound		
ECG		
CT Scan		
MRI		

Time allocation sheet

**Staff Member Code** (Enter Code as entered in Table 2):				
**Code for services**	**Activities that do not involve direct contact with single patient**	**Time per Patient (a)**	**Frequency[1] (b)**	**Average number of patients during each session i.e. review records or ask the staff (c)**
1.	*Outpatient Consultation*			
2.	*Inpatient care*			
3.	*Preparation/planning/dosimetry of 2DRT*			
4.	*Preparation/planning/dosimetry of 3DCRT*			
5.	*Preparation/planning/dosimetry of IMRT*			
6.	*Delivery of 2DRT*			
7.	*Delivery of 3DCRT*			
8.	*Delivery of IMRT*			
9.	*Quality assurance of machines*			
10.	*Teaching/training*			
11.	*Meetings*			

---

[1]***1' for once a year*** *participation, '**2' for twice a year**, **3 for thrice a year** participation, **4 for quarterly participation**, **5 for once every two months**, **6 for monthly participation**, **7 for fortnightly participation**, **8 for weekly participation**, **9 for twice a week participation**, **10 for thrice a week** participation, **11 for daily participation**.*
