# Supplementary material for: Cost of treatment for head and neck cancer in India
Source: PLoS One. 2018 Jan 11;13(1):e0191132. doi: 10.1371/journal.pone.0191132 (PMC5764364; doi:10.1371/journal.pone.0191132)
Supplement: S2 File — (PDF) [file pone.0191132.s003.pdf]

Cost effectiveness of IMRT versus Conventional Radiotherapy for the treatment of Head and Neck Cancers in India

GENERAL INFORMATION

C.R. No………………….. Patient Id*…/…/…….

RT Number…………

Date of Interview ----/----/---- Diagnosis: ……………

Type of treatment: …………….

Type of radiotherapy: …………….

1) Name of Patient …………………………......
2) Address of Patient

House No. ____

Sector/Street ____

City/State ____

Contact No.

Home_______________Office__________________Mobile_________________

Email id____

3) Gender

a) Male b) Female

4) Age of Patient (In completed years) ☐
5) Name of Respondent/Caregiver ……………………………………
6) Relation with Patient ……………………………………………….
7) Address of Respondent/Caregiver

House No. ____

Sector/Street ____

City/State ____

Contact No.

Home_______________Office__________________Mobile_________________

Email id____

8) Religion

a) Hindu | b) Muslim | c) Sikh
d) Christian | f) Others

9) Caste

a) Schedule caste | b) Schedule tribe | c) OBC
d) General | f) Others

10) Locality

a) Urban | b) Slum | c) Rural

11) Educational status

a) Illiterate | b) Primary | c) Middle
d) Matric | e) Senior secondary | f) Graduation
g) Post graduation

12) Occupation

a) Cultivator | b) Agricultural wage labourer
c) Non-agricultural wage labourer | d) Own account worker
e) Employer | f) Unpaid family worker
g) Regular salaried/Wage employee | h) Unemployed
i) Rentier/Pensioner/Other remittances recipient
j) Not able to work due to disability | k) Too old to work
l) Others

13) Marital Status

a) Unmarried | b) Married
c) Separated/Divorced | d) Widow/Widower

14) Type of Insurance

a) BPL free/poor free
b) Government employee
c) RSBY
d) Private Insurance
e) NRHM scheme
f) Any other, specify………
g) NO

15) Gross Monthly Income (Patient)……………………..Rs

16) Total family income ……………………….Rs

17) Total number of family members……………..

18) Total number of family members aged >10 years……………..

19) Total number of family members aged <10 years……………..

20) How many health facilities did you visit before coming to study hospital?

21) Where did you went, when you first came across the symptoms?

   a) Government facility, specify…………

   b) Private facility, specify…………..

   c) Any other specify…………..

22) When did you visit the above mentioned facility?............

23) Date of presentation at the study hospital? ……….

24) Date of start of radiotherapy treatment?...........

25) Date of end of radiotherapy treatment?...............

26) What was the duration of total treatment in the study hospital?...........

27) Any co morbidity…………

28) Previous history of

   a. Smoking

   b. Alcohol consumption

   c. Tobacco chewing

   d. Any other

29) Performance status after treatment (at the time of interview)…………

Tumour related Information:

**Tumour site**	*Oropharynx*	
	Hypopharynx	
	Supra-glottic region	
	Any other, specify	
**Tumour stage**	*Tis*	
	T1	
	T2	
	T3	
	T4a	
	T4b	
**Nodal stage**	*N0*	
	N1	
	N2	
	N2a	
	N2b	
	N2c	
	N3	
**Tumour stage**	*0*	
	I	
	II	
	III	
	Iva	
**WHO performance status**	*0*	Fully active, able to carry on all pre-disease performance without restriction
	1	Restricted in physically strenuous activity but ambulatory and able to carry out work of a light or sedentary nature, e.g., light house work, office work
	2	Ambulatory and capable of all selfcare but unable to carry out any work activities. Up and about more than 50% of waking hours
	3	Capable of only limited selfcare, confined to bed or chair more than 50% of waking hours
	4	Completely disabled. Cannot carry on any selfcare. Totally confined to bed or chair
	5	Dead

CONSUMPTION EXPENDITURE

How much does your family spend per month on following items:		Expense		
		7 days	30 days	365 days
i.	Food: ration (Cereals, pulses, edible oil, bread etc.), Fruits and vegetables, Milk, Milk products, Beverages etc			
ii.	Education (Books, newspaper, fees)			
iii.	Health			
iv.	Bills (Electricity, telephone, water)			
v.	Conveyance, fuel			
vi.	Rents			
vii.	Clothing, Footwear, bedding, curtains etc			
viii.	Entertainment (Cable, cinema, sports, recreation & hobbies)			
ix.	Personal effects (Watch, mobile phone, spectacles, toiletries, jewellery)			
x.	Consumer services (Domestic help, cook, sweeper, barber, tailor, priest, beautician)			
xi.	Pan, Tobacco, alcohol or any other intoxicants			
xii.	Miscellaneous (household appliances, furniture, crockery, animals, or any family function)			
xiii.	Total Monthly Expenditure (to be calculated from above)			

OUT OF POCKET EXPENDITURE

Expenditure before coming to the study hospital

Source of expenditure	Out of Pocket Expenditure
Travelling cost	
Medicines	
Lab tests/ Diagnostics	
Procedure/Surgery	
User fees/Hospital charges (including bed charges, diagnostics etc.)	
Informal payment	
Boarding/Lodging	
Food	
Other	
Total	

Source of finance

Source	Amount
Salary/Savings	
Selling of assets	
Borrowed from relatives/friends without interest	
Borrowed with interest	
Health insurance	
Any other (specify)	

Expenditure on Pre-Radiotherapy treatment at the study hospital

**Source of expenditure**	**Out of Pocket Expenditure**
Travelling cost	
Medicines	
Lab tests/ Diagnostics	
Procedure/Surgery	
User fees/Hospital charges (including bed charges, diagnostics etc.)	
Informal payment	
Boarding/Lodging	
Food	
Other	
Total	

Source of finance on pre radiotherapy treatment

Source	Amount
Salary/Savings	
Selling of assets	
Borrowed from relatives/friends without interest	
Borrowed with interest	
Health insurance	
Any other (specify)	

1. **Expenditure due to Radiotherapy treatment**

**Out of Pocket Expenditure**	**Week 1**						
	1	2	3	4	5	6	7
Travelling cost							
Medicines							
Lab tests/ Diagnostics							
Procedure/Surgery							
User fees/Hospital charges (including bed charges, diagnostics etc.)							
Informal payment							
Boarding/Lodging/Food							
Other1							
Total							

**Out of Pocket Expenditure**	**Week 2**						
	1	2	3	4	5	6	7
Travelling cost							
Medicines							
Lab tests/ Diagnostics							
Procedure/Surgery							
User fees/Hospital charges (including bed charges, diagnostics etc.)							
Informal payment							
Boarding/Lodging/Food							
Other1							
Total							

**Out of Pocket Expenditure**	**Week 3**						
	1	2	3	4	5	6	7
Travelling cost							
Medicines							
Lab tests/ Diagnostics							
Procedure/Surgery							
User fees/Hospital charges (including bed charges, diagnostics etc.)							
Informal payment							
Boarding/Lodging/Food							
Other1							
Total							

**Out of Pocket Expenditure**	**Week 4**						
	1	2	3	4	5	6	7
Travelling cost							
Medicines							
Lab tests/ Diagnostics							
Procedure/Surgery							
User fees/Hospital charges (including bed charges, diagnostics etc.)							
Informal payment							
Boarding/Lodging/Food							
Other1							
Total							

**Out of Pocket Expenditure**	**Week 5**						
	1	2	3	4	5	6	7
Travelling cost							
Medicines							
Lab tests/ Diagnostics							
Procedure/Surgery							
User fees/Hospital charges (including bed charges, diagnostics etc.)							
Informal payment							
Boarding/Lodging/Food							
Other1							
Total							

**Out of Pocket Expenditure**	**Week 6**						
	1	2	3	4	5	6	7
Travelling cost							
Medicines							
Lab tests/ Diagnostics							
Procedure/Surgery							
User fees/Hospital charges (including bed charges, diagnostics etc.)							
Informal payment							
Boarding/Lodging/Food							
Other1							
Total							

**Out of Pocket Expenditure**	**Week 7**						
	1	2	3	4	5	6	7
Travelling cost							
Medicines							
Lab tests/ Diagnostics							
Procedure/Surgery							
User fees/Hospital charges (including bed charges, diagnostics etc.)							
Informal payment							
Boarding/Lodging/Food							
Other1							
Total							

**Out of Pocket Expenditure**	**Week 8**						
	1	2	3	4	5	6	7
Travelling cost							
Medicines							
Lab tests/ Diagnostics							
Procedure/Surgery							
User fees/Hospital charges (including bed charges, diagnostics etc.)							
Informal payment							
Boarding/Lodging/Food							
Other1							
Total							

2. **Source of Finance**

Source	1st week	Amount	2nd week	Amount	3rd week	Amount	4th week	Amount
Salary/Savings								
Selling of assets								
Borrowed from relatives/friends without interest								
Borrowed with interest								
Health insurance								
Any other (specify)								

**Source; 1=Yes, 2=No*

Source	5th week	Amount	6th week	Amount	7th week	Amount
Salary/Savings						
Selling of assets						
Borrowed from relatives/friends without interest						
Borrowed with interest						
Health insurance						
Any other (specify)						

Expenditure on Chemotherapy/in-patient care in radiotherapy department

Source of expenditure	Out of Pocket Expenditure
Travelling cost	
Medicines	
Lab tests/ Diagnostics	
Procedure/Surgery	
User fees/Hospital charges (including bed charges, diagnostics etc.)	
Informal payment	
Boarding/Lodging	
Food	
Other	
Total	

Source of finance

Source	Amount
Salary/Savings	
Selling of assets	
Borrowed from relatives/friends without interest	
Borrowed with interest	
Health insurance	
Any other (specify)	

Data Collection Tool for Indirect expenditure due to treatment

Patient Details:

1. What would you have being doing otherwise if you were not taking treatment? (Multiple response allowed)

   Time spent (in hours) on:

	1 day	1 week	1 month
Household activities	____	____	____
Childcare	____	____	____
Professional work	____	____	____
Voluntary work	____	____	____
Leisure activities	____	____	____
Attending School/University	____	____	____
Seeking work	____	____	____
Social work	____	____	____
Physical workout	____	____	____
Other (specify)	____	____	____

2. Did other people take over and perform your usual household tasks during your hospital stay? If yes, fill the appropriate option, there can be more than one answer

	Yes/No/NA	Paid/Unpaid	No. of hours
Household activities	____	____	____
Childcare	____	____	____
Professional work	____	____	____
Voluntary work	____	____	____
Leisure activities	____	____	____
Attending School/University	____	____	____
Seeking work	____	____	____
Social work	____	____	____
Physical workout	____	____	____
Other (specify)	____	____	____

Caregivers:

	Caregiver 1	Caregiver 2	Caregiver 3
Relation with patient			
Address			
Contact No.			
No. of visits (per day)			
Total duration of Hospital stay (In hours)			
Employment status (Yes/No)			
Nature of employment (Give codes as mentioned in the end of tool)			
Monthly Gross Income of Caregiver (In Rs)			
**Time spent daily (hours) on:**			
**Household activities**			
Hours forgone due to care-giving			
Alternative {1=Yes(paid), 2=Yes (unpaid), 3=No, 4=NA}			
No. of hours (alternative)			
Payment to alternative paid worker (In Rs)			
**Childcare**			
Hours forgone due to care-giving			
Alternative {1=Yes(paid), 2=Yes (unpaid), 3=No, 4=NA}			
No. of hours (alternative)			
Payment to alternative paid worker (In Rs)			
**Professional work**			
Hours forgone due to care-giving			
Alternative {1=Yes(paid), 2=Yes (unpaid), 3=No, 4=NA}			
No. of hours (alternative)			
Payment to alternative paid worker (In Rs)			
**Voluntary work**			
Hours forgone due to care-giving			
Alternative {1=Yes(paid), 2=Yes (unpaid), 3=No, 4=NA}			

No. of hours (alternative)			
Payment to alternative paid worker (In Rs)			
**Leisure activities**			
Hours forgone due to care-giving			
**Attending School/university**			
Hours forgone due to care-giving			
**Seeking work**			
Hours forgone due to care-giving			
**Social work**			
Hours forgone due to care-giving			
Alternative {1=Yes(paid), 2=Yes (unpaid), 3=No, 4=NA}			
No. of hours (alternative)			
**Physical workout**			
Hours forgone due to care-giving			
**Other (specify)**			
Hours forgone due to care-giving			
Alternative {1=Yes(paid), 2=Yes (unpaid), 3=No, 4=NA}			
No. of hours (alternative)			

* *Alternative Worker;* Yes (Paid) =1, Yes (Unpaid) =2, No=3, Not Applicable (NA) =4

Employment Status; Cultivator=1, Agricultural wage labourer=2, Non-agricultural wage labourer=3, Own account worker=4, Employer=5, Unpaid family worker=6, Regular salaried/Wage employee=7, Unemployed=8, Rentier/Pensioner/Other remittance recipient=9, Not able to work due to disability=10, Too old to work=11, Other

Due to care-giving was your performance at work affected:

Care giving had no effect on my daily activities	0	1	2	3	4	5	6	7	8	9	10	Care giving stops my daily professional activities completely

(Encircle a number)

1. Due to care-giving was your performance on non-professional activities like household chores, shopping, exercising, studies etc. affected.

Care giving had no effect on my daily activities	0	1	2	3	4	5	6	7	8	9	10	Care giving stops my daily non-professional activities completely

(Encircle a number)

I am grateful to you for talking the time and making the effort to complete this questionnaire.

If you have any comments, remarks or suggestions, please let me know.

Signature/thumb impression of the respondent/s
